# 2-(2,4-Dioxy-1,2,3,4-Tetrahydropyrimidin-1-yl)-N-(4-Phenoxyphenyl)-Acetamides as a Novel Class of Cytomegalovirus Replication Inhibitors

**Published:** 2015

**Authors:** D. A. Babkov, M. P. Paramonova, A. A. Ozerov, A. L. Khandazhinskaya, R. Snoeck, G. Andrei, M. S. Novikov

**Affiliations:** Volgograd State Medical University, Pavshikh Bortsov Sq., 1, Volgograd 400131, Russia; Engelhardt Institute of Molecular Biology, Russian Academy of Science, Vavilov Str., 32, Moscow, 119991 , Russia; Rega Institute for Medical Research, KU Leuven, Minderbroedersstraat 10, Leuven B-3000, Belgium

**Keywords:** uracil derivatives, synthesis, antiviral activity, human cytomegalovirus

## Abstract

A series of novel uracil derivatives, bearing *N*-(4-phenoxyphenyl)acetamide moiety at N3 of a pyrimidine ring, has been synthesized. Their antiviral activity has been evaluated. It has been found that the novel compounds possess high inhibitory activity against replication of human cytomegalovirus (AD-169 and Davis strains) in HEL cell cultures. In addition, some of the derivatives proved to be inhibitory against varicella zoster virus.

## DISCUSSION

Cytomegalovirus (CMV) is widespread in the human population and has been found in people of all geographical regions as well as in representatives of all socio-economic groups [1]. CMV causes a lifelong latent infection that can reactivate periodically. In healthy individuals, the infection is usually asymptomatic [2]; however in individuals with reduced immune status, particularly in AIDS patients [3] and those receiving immunosuppressive therapy after organ transplantation [4], CMV is associated with significant morbidity and mortality. CMV is considered to be the most dangerous cause of congenital diseases. The virus can be transmitted from the mother to the fetus, resulting in a stillbirth, birth defects, and developmental disorders [5].

Ganciclovir, foscarnet, cidofovir and their prodrugs valganciclovir, and cidofovir are used to treat CMV [6]. However, these drugs cause many adverse side effects [6]. Long-term therapy of a CMV infection can lead to the emergence of resistant variants of CMV [7], therefore the search for new highly effective anti-CMV agents is an urgent task.

We have recently synthesized a number of 1-cinnamyl- 3-benzyl-uracil derivatives which effectively blocked the replication of HIV-1 and CMV in cell cultures [8], and we describe the synthesis and properties of 1-[ω-(phenoxy)alkyl]uracil derivative as an anti-CMV agent [9]. In the continuation of the search for new inhibitors of CMV replication, we synthesized uracil derivatives bearing *N*-(4-phenoxyphenyl)acetamide moiety at N3 of a pyrimidine ring and studied their antiviral properties.

**2-Chloro-N-(4-phenoxyphenyl)-acetamide (1)**

The suspension of 3.9 g (21.06 mmol) of 4-(phenoxy)- aniline (**2**) and 0.15 g of NH_4_Cl in 25 mL of HMDS was refluxed for 12 hours until a clear solution was obtained. The excess of HMDS was removed under reduced pressure, and 50 mL of anhydrous 1,2-dichloroethane was added to the residue (dark-colored oily liquid); then, 1.7 ml (21.37 mmol) of chloroacetyl chloride was added dropwise to the solution at 0 °C. The resulting mixture was stirred at 0 °C for 2 hours and allowed to stand overnight at room temperature. The reaction mixture was then evaporated under reduced pressure on a rotary evaporator and recrystallized from an ethyl acetatehexane (1:1) mixture. The resulting product was a light purple fine crystalline substance (80% yield), m.p. 105– 106 °C, R_f_ 0.62 (ethyl acetate-hexane, 1:1). 1H-NMR spectrum (DMSO-D_6_), δ, ppm, *J *(Hq): 4.24 (2H, s, COCH_2_), 6.97 (2H, d, *J *= 8.7, H-3’, H-5’), 7.00 (2H, d, *J *= 9.0, H-2’’, H-6’’), 7.10 (1H, t, *J *= 7.4, H-4’’), 7.36 (2H, t, *J *= 8.5, H-3’’, H-5’’), 7.61 (2H, d, *J *= 9.0, H-2’, H-6’), 10.30 (1H, s, NH). ^13^C-NMR-spectrum (DMSO-D_6_), δ, ppm: 47.7, 122.2, 123.6, 125.4, 134.2, 138.5, 156.6, 161.4, 168.7.

**General procedure for the synthesis of 2-(2,6-dioxy-3,6-dihydropyrimidin-1(2H)-yl)- N-(4-phenoxyphenyl)acetamides (4)-(11)**

A mixture of 1.42 mmol of the appropriate 1-substituted- uracil (**12**)-(**19**) and 0.29 g (2.10 mmol) K_2_CO_3_ in 10 mL of DMF solution was stirred at 80 °C for 1 h, cooled to room temperature, and 2.12 mmol of 2-chloro-N-(4-phenoxyphenyl)acetamide (**1**) was added to the mixture; the reaction mixture was stirred at the same temperature for 24 hours. Then the reaction mixture was filtered, evaporated *in vacuo, *and purified by flash chromatography, followed by recrystallization of the product from ethyl acetate-hexane (1:1).

**2-(3-Benzyl-2,6-dioxo-3,6-dihydropyrimidin- 1(2H)-yl)-N-(4-phenoxyphenyl)acetamides (4)**

Yield 85%, m.p. 186–187°C, R_f_ 0.60 (1,2-dichloroethane– ethyl acetate, 1:1). 1H-NMR-spectrum (DMSO-D_6_) δ, ppm, *J *(Hz): 4.21 (2H, s, CH_2_), 4.53 (2H, s, CH_2_), 5.38 (1H, d, *J *= 7.8, H-5), 6.51–6.56 (4H, m, H-4’, H-3’, H-5’, H-4’’’), 6.65 (2H, d, *J *= 8.5, H-2’, H-6’), 6.84–6.94 (6H, m, H-3’’, H-5’’, H-2’’’, H-3’’’, H-5’’’, H-6’’’), 7.15 (2H, d, *J* = 8.9, H-2’’, H-6’’), 7.43 (1H, d, *J *= 7.8, H-6), 9.88 (1H, s, NH). ^13^C-NMR-spectrum (DMSO-D_6_), δ, ppm: 42.9, 51.0, 100.1, 117.5, 119.1, 120.3, 122.6, 127,1, 127.4, 128.3, 129.5, 134.3, 136.1, 144.1, 150.8, 151.4, 156.9, 161.8, 164.7.

**2-[3-(4-Methylbenzyl)-2,6-dioxo-3,6- dihydropyrimidin-1(2H)-yl]-N-(4- phenoxyphenyl)acetamide (5)**

Yield 83%, m.p. 193–194°C, R_f_ 0.54 (1,2-dichloroethane– ethyl acetate, 1:1). 1H-NMR-spectrum (DMSO-D_6_) δ, ppm, *J *(Hz): 2.29 (3H, s, CH_3_), 4.65 (2H, s, CH_2_), 4.92 (2H, s, CH_2_), 5.80 (1H, d, *J *= 7.8, H-5), 6.98 (2H, d, *J *= 8.5, H-3’, H-5’), 7.00 (2H, d, *J *= 8.9, H-2’, H-6’), 7.10 (1H, dt,* J *= 7.3 and 1.0, H-4’’’), 7.19 (2H, d, *J *= 7.9, H-2’’, H-6’’), 7.23 (2H, d, *J *= 7.8, H-3’’, H-5’’), 7.36 (2H, dt, *J *= 7.4 and 1.2, H-3’’’, H-5’’’), 7.60 (2H, d, *J *= 8.8, H-2’’’, H-6’’’), 7.84 (^1^H, d, *J *= 8.0, H-6), 10.25 (^1^H, s, NH). 13C-NMRspectrum (DMSO-D_6_), δ, ppm: 24.9, 47.5, 55.4, 104.7, 122.2, 123.7, 125.0, 127.2, 131.8, 133.4, 134.2, 137.7, 138.9, 141.3, 148.6, 155.4, 156.1, 161.6, 166.4, 169.3.

**2-[3-(3,5-Dimethylbenzyl)-2,6-dioxo- 3,6-dihydropyrimidin-1(2H)-yl]-N-(4- phenoxyphenyl)acetamide (6)**

Yield 79%, m.p. 99–101°C, R_f_ 0.53 (1,2-dichloroethane– ethyl acetate, 1:1). ^1^H-NMR-spectrum (DMSO-D_6_) δ, ppm, *J *(Hz): 2.24 c (6H, CH_3_), 4.64 c (2H, CH_2_), 4.87 c (2H, CH_2_), 5.80 d (1H, *J *= 7.9, H-5), 6.92 c (3H, H-2’, H-4’, H-6’), 6.96 d (2H, *J *= 8.0, H-2’’, H-6’’), 6.98 d (2H, *J *= 8.9, H-3’’, H-5’’), 7.09 t (^1^H, *J *= 7.3, H-4’’’), 7.35 t (2H, *J* = 7.8, H-3’’’, H-5’’’), 7.58 d (2H, *J *= 8.8, H-2’’’, H-6’’’), 7.82 d (^1^H, *J *= 7.9, H-6), 10.29 c (^1^H, NH). ^13^C-NMRspectrum (DMSO-D_6_), δ, ppm: 25.1, 47.5, 55.6, 104.7, 122.1, 123.7, 124.9, 127.2, 129.5, 133.4, 134.2, 138.9, 140.5, 142.0, 148.7, 155.4, 156.1, 161.5, 166.4, 169.3.

**2-(3-Cinnamyl-2,6-dioxo-3,6-dihydropyrimidin- 1(2H)-yl)-N-(4-phenoxyphenyl)acetamide (7)**

Yield 88%, m.p. 184–185°C, R_f_ 0.41 (1,2- dichloroethane– ethyl acetate, 1:1). 1H-NMR-spectrum (DMSO-D_6_) δ, ppm, *J *(Hz): 4.54 (2H, d, *J *= 5.6, CH_2_), 4.64 (2H, s, CH_2_), 5.81 (^1^H, d, *J *= 8.0, H-5), 6.34 (^1^H, dt, *J *= 6.0, =CH-), 6.60 (^1^H, d, *J *= 16.0, PhCH=), 6.96 (2H, d, *J *= 7.8, H-3’’, H-5’’), 6.98 (2H, d, *J *= 8.9, H-2’’, H-6’’), 7.09 (^1^H, t, *J *= 7.3, H-4’’’), 7.25 (^1^H, t, *J *= 7.4, H-4’), 7.33 (2H, t, *J *= 7.8, H-3’’’, H-5’’’), 7.35 (2H, t, *J *= 8.4, H-3’, H-5’), 7.43 (2H, d, *J *= 7.5, H-2’, H-6’), 7.58 (2H, d, *J *= 8.9, H-2’’’, H-6’’’), 7.77 (1H, d, *J *= 7.8, H-6), 10.28 (^1^H, s, NH). ^13^C-NMRspectrum (DMSO-D_6_), δ, ppm: 47.4, 54.2, 104.7, 122.1, 123.7, 125.0, 127.2, 128.0, 130.7, 132.2, 132.9, 134.2, 137.0, 138.9, 140.1, 148.4, 155.2, 156.1, 161.5, 166.5, 169.3.

**2-[3-(Napht-1-ylmethyl)-2,6-dioxo- 3,6-dihydropyrimidin-1(2H)-yl]-N-(4- phenoxyphenyl)acetaminde (8)**

Yield 85%, m.p. 197–198.5°C, R_f_ 0.57 (1,2-dichloroethane– ethyl acetate, 1:1). ^1^H-NMR-spectrum (DMSO-D_6_) δ, ppm, *J *(Hz): 4.72 (2H, s, CH_2_), 5.49 (2H, s, CH_2_), 5.85 (^1^H, d, *J *= 8.0, H-5), 6.99 (2H, d, *J *= 7.8, H-3’’, H-5’’), 7.02 (2H, d, *J *= 9.0, H-2’’, H-6’’), 7.11 (^1^H, t, *J *= 7.3, H-4’’’), 7.36 (^1^H, d, *J *= 7.1, H-4’), 7.37 (2H, dt, *J *= 8.6 and 0.9, H-3’’’, H-5’’’), 7.51 (^1^H, t, *J *= 7.9, H-6’), 7.57– 7.63 (4H, m, H-3’, H-7’, H-2’’’, H-6’’’), 7.76 (^1^H, d, *J* = 7.8, H-6), 7.92 (^1^H, d, *J *= 8.3, H-8’), 7.99 (^1^H, d, *J *= 7.7, H-4’), 8.12 (1H, d, *J *= 8.2, H-5’), 10.28 (1H, s, NH). 13C-NMR-spectrum (DMSO-D_6_), δ, ppm: 47.6, 53.2, 105.1, 122.2, 123.7, 125.0, 127.2, 129.1, 129.7, 130.4, 130.9, 132.5, 132.9, 134.2, 134.6, 136.1, 137.6, 138.9, 148.3, 155.6, 156.2, 161.6, 166.4, 169.3.

**2-[3-[2-(Phenoxy)ethyl]-2,6-dioxo- 3,6-dihydropyrimidin-1(2H)-yl]-N-(4- phenoxyphenyl)acetamide (9)**

Yield 90%, m.p. 154–155°C, R_f_ 0.46 (1,2-dichloroethane– ethyl acetate, 1 : 1). ^1^H-NMR-spectrum (DMSO-D_6_) δ, ppm, *J *(Hz): 4.16 (2H, d, *J *= 5.4, NCH_2_), 4.21 (2H, d, *J *= 5.4, OCH_2_), 4.64 (2H, s, CH_2_), 5.78 (^1^H, d, *J *= 8.0, H-5), 6.94–7.00 (7H, m, H-2’, H-4’, H-6’, H-2’’, H-3’’, H-5’’, H-6’’), 7.10 (^1^H, t, *J *= 7.3, H-4’’’), 7.29 (2H, t, *J *= 7.9, H-3’’’, H-5’’’), 7.36 (2H, t, *J *= 7.6 and 1.2, H-3’, H-5’), 7.59 (2H, d, *J *= 8.9, H-2’’’, H-6’’’), 7.78 (^1^H, d, *J *= 7.9, H-6), 10.24 (^1^H, s, NH). ^13^C-NMR-spectrum (DMSO-D_6_), δ, ppm: 29.7, 47.4, 52.4, 69.4, 104.0, 118.9, 122.2, 123.7, 125.0, 125.3, 127.2, 133.8, 134.1, 138.9, 149.3, 155.4, 156.2, 161.6, 162.2, 166.4, 169.3.

**2-[3-(Benzyloxymethyl)-2,6-dioxo- 3,6-dihydropyrimidin-1(2H)-yl]-N-(4- phenoxyphenyl) acetamide (10)**

Yield 84%, m.p. 163–164°C, R_f_ 0.47 (1,2-dichloroethane– ethyl acetate, 1 : 1). ^1^H-NMR-spectrum (DMSO-D_6_) δ, ppm, *J *(Hz): 4.59 (2H, s, CH_2_), 4.63 (2H, s, CH_2_), 5.26 (2H, s, CH_2_), 5.83 (^1^H, d, *J *= 7.8, H-5), 6.96 (2H, d, *J* = 7.9, H-3’’, H-5’’), 6.99 (2H, d, *J *= 8.9, H-2’’, H-6’’), 7.09 (^1^H, dt, *J *= 7.6 and 1.0, H-4’’’), 7.26–7.38 (7H, m, C6H’5, H-3’’’, H-5’’’), 7.59 (2H, d, *J *= 9.1, H-2’’’, H-6’’’), 7.84 (^1^H, d, *J *= 8.0, H-6), 10.32 (^1^H, s, NH). ^13^C-NMRspectrum (DMSO-D_6_), δ, ppm: 43.2, 70.4, 77.2, 100.9, 117.9, 119.5, 120.7, 123.0, 127.69, 127.74, 128.3, 130.0, 134.7, 137.4, 143.8, 151.3, 151.9, 157.4, 162.1, 165.1.

**2-(3-Propargyl-2,6-dioxo-3,6-dihydropyrimidin- 1(2H)-yl)-N-(4-phenoxyphenyl)acetamide (11).**

Yield 81%, m.p. 226–228°C, R_f_ 0.68 (1,2- dichloroethane– ethyl acetate, 1:1). ^1^H-NMR-spectrum (DMSO-D_6_) δ, ppm, *J *(Hz): 3.42 (1H, s, ≡CH), 4.59 (2H, s, CH_2_), 4.60 (2H, d, *J *= 8.0, CH_2_), 5.81 (1H, d, *J *= 8.0, H-5), 6.95 (2H, d, *J *= 7.7, H-3’’, H-5’’), 6.98 (2H, d, *J *= 8.9, H-2’’, H-6’’), 7.08 (1H, t, *J *= 7.6, H-4’’’), 7.35 (2H, dt, *J *= 8.5 and 1.1, H-3’’’, H-5’’’), 7.56 (2H, d, *J *= 8.9, H-2’’’, H-6’’’), 7.80 (1H, d, *J *= 7.9, H-6), 10.33 (1H, s, NH). 13C-NMRspectrum (DMSO-D_6_), δ, ppm: 42.0, 47.4, 80.3, 82.4, 105.1. 122.1, 123.7, 125.0, 127.3, 134.2, 138.8, 147.6, 154.8, 156.1, 161.5, 166.3, 169.2.

**Antiviral research**

Activity of the compounds was evaluated against the following viruses: thymidine kinase deficient (TK-) herpes simplex virus type 1 (HSV-1) KOS strain, HSV- 1 KOS strain resistant to acyclovir (ACVr), herpes simplex virus type 2 Lyons and G strains, CMV (AD-169 and Davis strains), varicella-zoster (VZV, OKA and YS strains), vaccinia virus Lederle strain, respiratory syncytial virus (Long strain), vesicular stomatitis virus, Coxsackie virus B4, parainfluenza virus 3, influenza A (sub-types H1N1, H3N2), influenza virus B, reovirus-1 virus, Sindbis virus, and Punta Toro virus. Investigations were carried out as described in [9].

2-Chloro-N-(4-phenoxyphenyl)acetamide (**1**) was synthesized as described previously [10]. The uracil derivatives substituted at N1 (**12**)–(**19**) were obtained by condensation of equimolar amounts of 2,4-bis- (trimethylsilyloxy)-pyrimidine and arylmethylchloride/ bromide as described in [8]. The treatment with equimolar amount of the chloride (**1**) in DMF in the presence of K_2_CO_3_, as shown, resulted in the target 4-phenoxyacetanelides (**4**)- (**11**) with 79-90% yields.

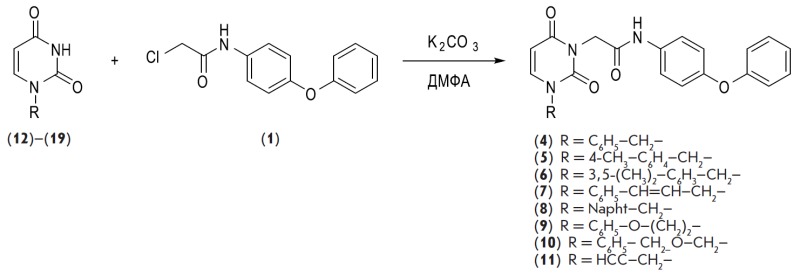



Anti-CMV properties of the uracil derivatives (**4**)- (**11**) were studied in HEL cell culture against CMV (AD-169 and Davis strains). It has been found that certain compounds of this series exhibit strong inhibitory activity against CMV, which is comparable with the effect of ganciclovir. The uracil derivatives substituted at position 1 of the pyrimidine ring with benzyl (compound (**4**)) and 3,5-dimethylbenzyl (compound (**6**)) were the most active. They inhibited CMV replication with EC50 = 3.06-8.9 μM. Other modifications of the structure resulted in complete loss of inhibitory activity.

It has also been found that the compounds (**4**) and (**6**) exhibit significant activity against VZV. They blocked VZV replication (Oka strain) in a HEL cell culture with EC50 = 8.18 μM (compound (**4**)) and 17.0 μM (compound (**6**)), which is inferior to the protective action of acyclovir (EC50 = 1.33 μM) and brivudine (EC50 = 0.026 μM), currently used to treat infections caused by this virus [11]. However, the thymidine kinase deficient VZV mutant strain (07-1), which is resistant to acyclovir and brivudine, was susceptible to 1-benzyl-3- acetanilide uracil derivatives with EC50 = 6.68 μM (compound (**4**)) and 16.1 μM (compound (**6**)).

Therefore, the uracil derivatives whose synthesis is described in this work represent a new class of inhibitors of CMV reproduction whose effect is comparable to that of ganciclovir. Furthermore, some compounds of this series have pronounced inhibitory effect on VZV, both the wild-type strain (OKA) and the strain (07-1) resistant to the action of acyclovir. The data demonstrate that it is a promising direction for the development of new effective antiviral agents.

## Abbreviations

HIV: human immunodeficiency virus
CMV: cytomegalovirus
AIDS: acquired immunodeficiency syndrome
HMDS: hexamethyldisilazane
DMSO: dimethyl sulfoxide
DMF: N,N-dimethylformamide
VZV: varicella zoster virus
